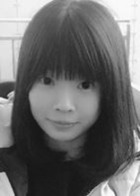# Electrochemical Quantification of Neurotransmitters in Single Live Cell Vesicles Shows Exocytosis is Predominantly Partial

**DOI:** 10.1002/cbic.202000622

**Published:** 2020-11-11

**Authors:** Ying Wang, Andrew Ewing

**Affiliations:** ^1^ Department of Chemistry and Molecular Biology University of Gothenburg Kemivägen 10 412 96 Gothenburg Sweden

**Keywords:** electrochemical cytometry, exocytosis, open and closed mechanism, partial release, vesicles

## Abstract

Exocytosis plays an essential role in the communication between cells in the nervous system. Understanding the regulation of neurotransmitter release during exocytosis and the amount of neurotransmitter content that is stored in vesicles is of importance, as it provides fundamental insights to understand how the brain works and how neurons elicit a certain behavior. In this minireview, we summarize recent progress in amperometric measurements for monitoring exocytosis in single cells and electrochemical cytometry measurements of vesicular neurotransmitter content in individual vesicles. Important steps have increased our understanding of the different mechanisms of exocytosis. Increasing evidence is firmly establishing that partial release is the primary mechanism of release in multiple cell types.

## Graphical abstract

**Partial release mechanism of exocytosis**: This minireview highlights the recent progress in amperometric measurement for monitoring exocytosis from single cells, and electrochemical cytometry measurement of vesicular neurotransmitter content in individual vesicles. Evidence from several studies supports the hypothesis that partial release is the primary mechanism during regular exocytosis.

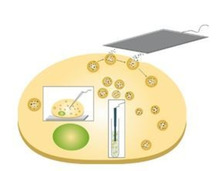

## 1 Introduction

The nervous system is composed of billions of neurons and efficient communication between neurons is crucial to maintain the functioning of the brain. Neurotransmission is the fundamental process that transfers information between neurons. Neurotransmission occurs at the synapse, where controlled amounts of neurotransmitters are released by exocytosis from synaptic vesicles. Vesicles are small, typically ranging from 50 to 800 nm in diameter.^[1]^ Modulation of synaptic exocytosis is considered to drive cognitive processes, including learning and memory.[^2^, ^3^] Thus, it is important to understand the mechanism of how neurotransmitters are released during exocytosis and their storage in individual vesicles.

It has been long debated whether exocytosis is an all‐or‐none process, often referred to as full release. In the all‐or‐none hypothesis, attributed to Katz,[^4^, ^5^] the vesicle membrane is assumed to fully distend into the plasma membrane, resulting in an irreversible fusion pore opening and all vesicular content is released. In contrast to the full fusion exocytosis, mathematical models of the initial fusion pore size were studied extensively by Amatore and co‐workers,[^6^, ^7^, ^8^] suggesting that the final pore opening angle is only a few tens of degrees at the maximum and thus, full opening of the fusion pore is very unlikely. Other exocytosis mechanisms, kiss‐and‐run as well as open and closed, have been later identified.^[1]^ In the kiss‐and‐run mechanism, the vesicle fuses with the plasma membrane to transiently form a small fusion pore with a diameter of 2–4 nm that only allows a small fraction of vesicle content to release.[^9^, ^10^, ^11^, ^12^] The vesicle then rapidly closes and is retrieved to be reloaded with further neurotransmitters. An open and closed mechanism, also known as partial release, has been recently proposed and suggested to be the primary mechanism during general exocytotic process and is now thought to represent the vast majority of what were originally thought to be full exocytosis events.[^1^, ^13^] Thus, full release involves the full distention of the vesicle, kiss‐and‐run involves only very short opening time and subsequent release, and partial release involves a further opening, wider than 2–4 nm in kiss‐and‐run, resulting in a larger fraction of vesicular content being released in the normal and most frequent mode of exocytosis. Partial release can result in a variable amount of the vesicular content being released. In this mode, the vesicle is then re‐used until perhaps losing a key component at which time it is recycled.

**Figure 1:**
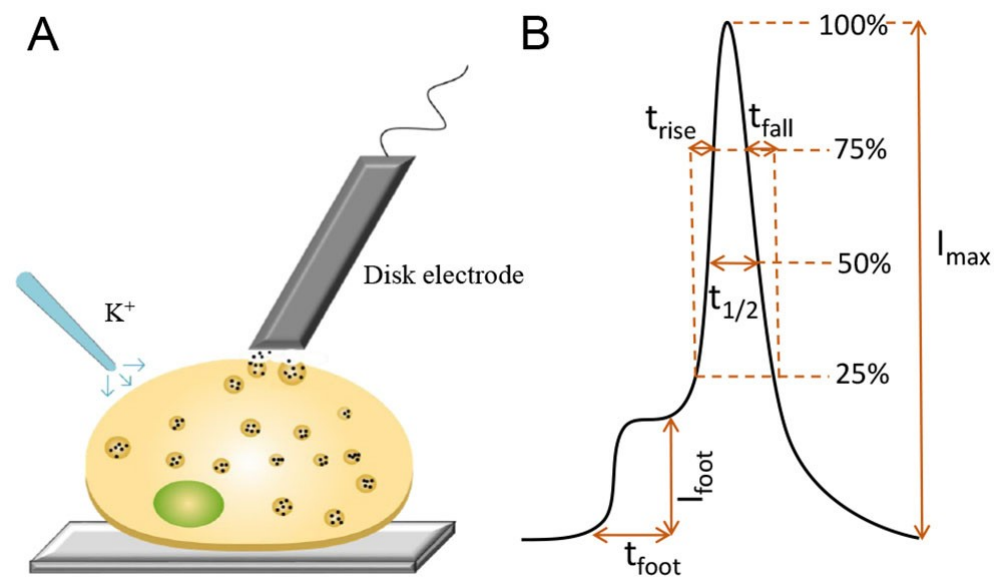
A) Single‐cell amperometry (SCA). B) Scheme of different parameters that are used for the peak analysis of exocytosis.

Amperometry at carbon‐fiber micro or nanoelectrodes is a powerful electrochemical technique that is used to quantify neurotransmitters release. It has high sensitivity, allowing real time quantification of neurotransmitters release from individual vesicles, and high temporal resolution, providing information of the release kinetics of exocytosis. Single‐cell amperometry was introduced by the Wightman group to detect exocytosis from single adrenal medullary chromaffin cells in 1990.^[14]^ In single cell amperometry (SCA), a carbon‐fiber microelectrode is placed in close proximity to a cell at a constant potential that is sufficient to oxidize electroactive neurotransmitters in a diffusion‐limited rate (Figure 1A). The current spikes generated from the electrochemical reaction during exocytosis allows quantification of neurotransmitters according to Faraday's law (*Q*=*nNF*), where *Q* is the total charge transferred, *n* is the number of exchanged electron in the reaction, *N* is the number of moles of neurotransmitters detected from the vesicle, and *F* is the Faraday constant, 96485 C/mol. The characteristics of each single current spike can also be used to determine dynamic information about an exocytotic event (Figure 1B). The current spike rise time corresponds to the opening of the fusion pore and the half width of spike represents the duration of the exocytotic event. Pre spike feet features were observed by the Neher group in 1992, where most of current spikes are preceded by a small foot signal, indicating a slow leakage of neurotransmitters during the early formation of the fusion pore.^[15]^

The focus of this minireview is to summarize and highlight some of the most recent advances in single vesicle quantification for monitoring exocytosis of chemical messengers, focusing on those that are organic small molecules. Some recent work examining other species, especially reactive oxygen and nitrogen species has been published recently and could be the subject of an entire review in itself.[^16^, ^17^, ^18^, ^19^, ^20^] Here we focus on work that has ultimately led to intracellular measurements of vesicle content. The combination of electrochemical cytometry and single‐cell amperometry at micro or nanoelectrodes in exocytosis analysis in different cell types and representative studies will be discussed. The effects of drug treatment and other factors on fusion pore dynamics as well as storage of neurotransmitters in single vesicles will also be reviewed. All findings to date suggest that partial release is the primary mechanism during regular exocytosis.

## 2 Electrochemical Cytometry To Measure Vesicular Content

Single‐cell amperometry provides information on the amount of neurotransmitters during exocytotic release, but to determine the fraction released, the quantity of neurotransmitters stored in a single vesicle is also needed. Flow electrochemical cytometry, a combination of amperometry and flow cytometry, initially allowed determination of neurotransmitter storage in nanometer vesicles.^[21]^

### 2.1 Flow vesicle electrochemical cytometry (FVEC)

**Figure 2:**
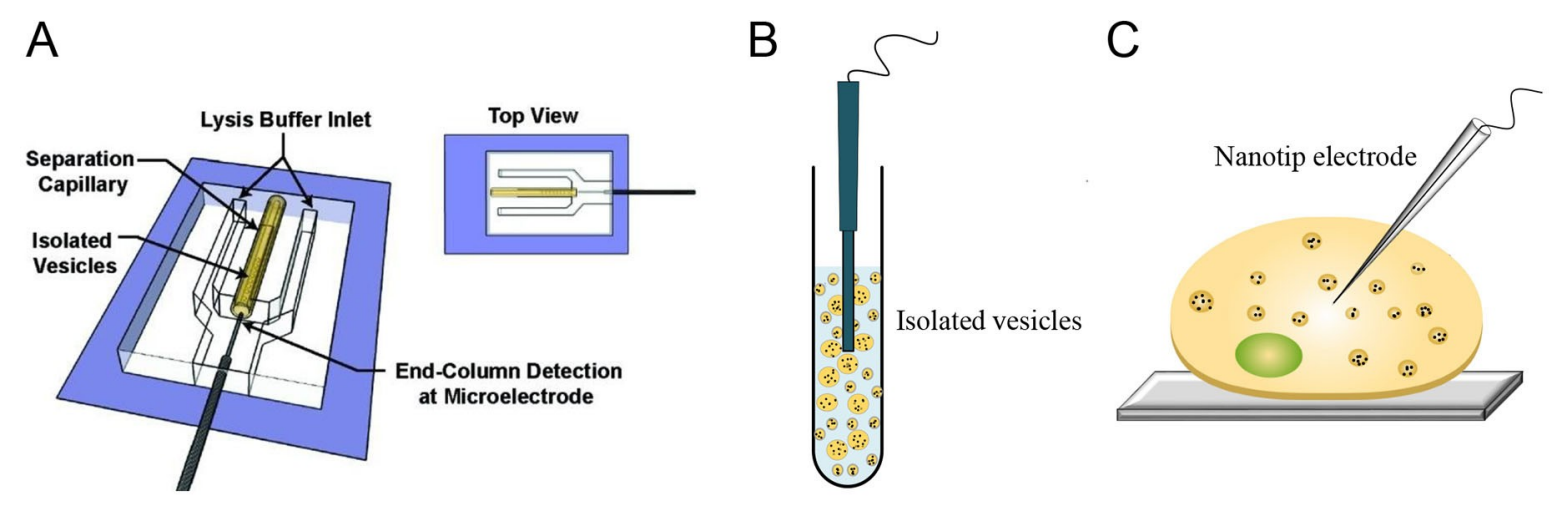
Electrochemical cytometry methods to measure vesicular content. A) Flow vesicle electrochemical cytometry (FVEC). Adapted with permission from ref. [21]. Copyright: 2010, American Chemical Society. B) Vesicle impact electrochemical cytometry (VIEC). C) Intracellular vesicle impact electrochemical cytometry (IVIEC).

The original flow‐based electrochemical cytometry was designed to quantify vesicular neurotransmitter content by combining capillary electrophoresis, microfluidics, and electrochemistry (Figure 2A).^[21]^ In flow vesicle electrochemical cytometry (FVEC), individual vesicles were isolated from a suspension of vesicles through capillary electrophoresis and then entered into a microfluidic device, where they were flushed with a sheath‐flow of surfactant solution to lyse their membrane, so all contents of vesicle could be released and directly detected by amperometry at carbon‐fiber microelectrodes. In those experiments, comparing SCA to FVEC, it was found that the average vesicle only released about 40 % of the total catecholamine during exocytotic release in PC12 cells. About 33 000 dopamine molecules presented per vesicle in mouse striatal were detected by FVEC, while the amount of transmitter content during exocytotic release is lower at cultured neurons.^[22]^ These findings lead to further experiments to determine that only a fraction of the vesicular content is released and this fraction can vary widely.

### 2.2 Vesicle impact electrochemical cytometry (VIEC)

Vesicle impact electrochemical cytometry (VIEC) was then developed to eliminate the separation step and simplify quantification of neurotransmitter content in isolated vesicles.^[23]^ In this method, a disk‐shaped carbon‐fiber electrode was placed in a suspension of isolated vesicles (Figure 2B). Vesicles adsorb on the surface of the electrode and rupture by electroporation,^[24]^ resulting in the opening of a pore on the vesicle membrane and subsequent release of electroactive messenger molecules. These released messengers are oxidized at the electrode surface, where they are restricted from diffusing away from the electrode and thus the total content can be quantified. The pore opening of vesicles on the electrode is potential dependent, whereas the number of molecules per vesicle is not affected.^[24]^ It is hypothesized that proteins on the membrane of the vesicle act as a barrier between the membrane and the electrode reducing the electroporation field and consequently these must move, possibly by random motion, prior to vesicle opening.^[24]^ Vesicle rupture on the electrode surface is also temperature and vesicle size dependent.^[25]^ Increasing the temperature from 6 to 30 °C facilitates electroporation‐induced pore formation and it is easier for larger vesicles rupture on the electrode than the smaller vesicles, consistent with the need for proteins to move and allow direct contact of the membrane lipids with the electrode. Fluorescence labeling of vesicles also facilitates vesicle rupture by electroporation.^[26]^ It was shown that a light‐stimulated fluorophore, rhodamine phosphatidylethanolamine or benzoxadiazole‐phosphoethanol‐amine, attached to the membrane of vesicle increases the number of amperometric events, corresponding to an increase in vesicle opening. This has been hypothesized to occur via production of reactive oxygen species by excited fluorophores causing oxidation of the membrane lipids and proteins and therefore, changing the conformation of the membrane of vesicle to allow easier adsorption at the electrode surface.

### 2.3 Intracellular vesicle impact electrochemical cytometry (IVIEC)

Intracellular vesicle impact electrochemical cytometry (IVIEC) was recently introduced to directly quantify vesicular content inside a single cell (Figure 2C).^[27]^ A cylindrical carbon‐fiber microelectrode was flame‐etched to obtain a thin needle shape with 50–100 nm tip diameter and tens of micrometer long. This nanotip electrode can be used to penetrate the cell membrane localizing in the cytoplasm of a live cell with minimal damage. This provides better sensitivity, dynamics, signal‐to‐noise ratio, and faster time response for many messengers detected in comparison of electrochemically etched as well as regular cylindrical‐shaped carbon‐fiber microelectrodes. Similar to the VIEC method, IVIEC is based on the same principles as VIEC, where the intracellular vesicles are adsorbed on the electrode surface and rupture by electroporation to release their contents.

### 2.4 VIEC versus IVIEC

Quantitative modeling of collection efficiencies from carbon‐fiber microelectrodes demonstrates that almost 100 % of vesicular content is captured and oxidized on a disk‐shaped carbon‐fiber electrode in VIEC, regardless of the location of the release pore.^[28]^ The collection efficiency of nanotip conical electrodes is dependent on the position of the vesicular release pore in IVIEC, where 75 % of vesicular content is predicted to be collected when the release pore is opposite to the electrode surface and 100 % can be captured when the release pore is close or at the electrode surface, but overall this approach provides reliable measurement of vesicular content.

The VIEC and IVIEC methods can be utilized for different purposes. For example, physical size and vesicular content of a single vesicle can be simultaneously measured by combining resistive pulse measurements and VIEC.^[29]^ Here, a nanopore pipet was used to eject single vesicles with different osmolality of solution inside and outside of the nanopipette tip by applying periodic pressure. A resistive pulses was generated when the vesicle was pushed through the pore. The vesicle then adsorbed on the surface of a carbon‐fiber electrode opens to release its content by electroporation and low osmolarity of the surrounding solution. However, depending on the cell type, the problem of adequate vesicle isolation remains a challenge. In addition, the vesicular catecholamine content quantified with VIEC is lower than that of IVIEC, as the vesicular neurotransmitter content in isolated vesicles decreases with higher speed centrifugation force during isolation steps.^[30]^ IVIEC with a nanotip electrode can directly assess vesicular content in a single living cell under various stimulations or drug treatments, allowing direct comparison to exocytotic release. Here, an advantage is that vesicle isolation is not required.

## 3 Electrochemical Detection of Vesicular Exocytosis

To examine the release mechanisms of exocytosis, the combination of SCA and IVIEC or VIEC have been used. The ability to quantify the amount of neurotransmitter released during exocytosis by SCA and storage in vesicles, by VIEC or IVIEC, make it possible to gain novel insights into both the fraction of release and regulation of this fraction to alter exocytosis.

### 3.1 Exocytosis of catecholamines in different cell types

Pheochromocytoma (PC12) cells and adrenal chromaffin cells are widely used as model cell lines for the study of neuronal secretion. These cells serve as useful models to investigate the fundamental exocytotic process and examine the key factors that influence exocytosis.

Zinc is an essential trace element in the brain and plays a crucial role in synaptic plasticity, learning and memory. Zinc treatment in PC12 cells significantly decreased vesicular catecholamine content.^[31]^ A larger pore opening during exocytosis was hypothesized leading to a higher fraction of catecholamine released in comparison to the control cells (92 vs. 66 %). The substructure of the vesicle should at least in part regulate the kinetics of the exocytotic process.^[32]^ Transmission electrode microscopy (TEM) has revealed that the vesicular volume decreases after zinc incubation, where the vesicular dense core expands and the halo space decreases in volume. This change of vesicular volume indicates that the amount of catecholamine stored in the fast‐releasing pool is decreased and in the slow‐releasing pool is increased, which explains why the fusion pore opens longer during exocytosis. The effect of zinc on vesicular storage is reversible.^[33]^ Post treatment with TPEN, a membrane‐permeable zinc chelator, in zinc‐treated cells leads to a reversible change by zinc in vesicular storage, but the effect of zinc on exocytotic release appears to be irreversible. Thus, the effect of zinc on vesicular storage and exocytotic release could be important for the formation and storage of memory.

ATP is the main energy source for all cellular processes and an excitatory neurotransmitter regulating the activities of neurotransmitters. Incubation of chromaffin cells with ATP increases exocytotic release apparently by resulting in a longer fusion pore expansion. The increase in rise time was dependent on the ATP concentration and a longer duration of pre‐spike feet was observed.^[34]^ The effect of ATP on exocytotic release is regulated by purinergic receptors. IVIEC was used to show that vesicular content remains unchanged, but the fraction of catecholamine released during exocytosis was increased with ATP treatment. ATP also acts as an energy source for the loading of vesicular content. Incubation with ATP and norepinephrine significantly increased both exocytotic release and vesicular content of catecholamine and it was concentration dependent.^[35]^ However, incubation with norepinephrine alone did not affect exocytosis or vesicular content, indicating additional neurotransmitter loading requires energy to increase the content that is stored in vesicles. Thus, ATP could act as a neurotransmitter to increase exocytotic release through purinergic receptors or as the energy source for the loading of vesicular content.

General anesthetics are essential medicines and have a marked effect on synaptic transmission. Barbiturate treatment alters exocytotic release in PC12 cells, resulting in fewer molecules released during exocytosis.^[36]^ Treatment with barbiturate apparently causes the formation of an unstable fusion pore, thus making the process of vesicle opening and closing faster. IVIEC was used to show that the vesicular content is not affected by barbiturate, but the fraction of catecholamine release during exocytosis is decreased. Local anesthetics, for example lidocaine, affect exocytosis in a concentration dependent way.^[37]^ The amount of catecholamine released during exocytosis increases at lower concentrations of lidocaine treatment (<0.1 mM), where the fusion pore opens longer with more release. However, at higher concentrations of lidocaine, lidocaine has an inhibitory effect, leading to a longer duration of the pore opening but less release of catecholamine. These results provide fundamental insights into how anesthetics affect cell function at the single‐cell level.

SCA and IVIEC have also been critical to reveal the function of drugs of abuse on exocytosis. The psychostimulant drugs, cocaine and methylphenidate, exhibit different effects on exocytosis and the fraction of catecholamine release.^[38]^ Cocaine and methylphenidate both significantly decreased exocytotic release and vesicular storage of catecholamine in PC12 cells, but reveal some important differences. In cocaine‐treated cells, the pore opening and closing times are shorter, but stay unchanged in methylphenidate‐treated cells. Cocaine and methylphenidate, however, have opposite effects on the fraction of release during exocytosis, where the fraction of release decreases to 65 % after cocaine (from 74 % in the control cells) and increases to 83 % for methylphenidate‐treated cells. These fundamental results may help to develop effective treatment with drug addiction, however it is interesting to note that these drugs have opposite effects on cognition,^[39]^ leading to speculation that the fraction released might be important in plasticity or the initiation of memory.

**Figure 3:**
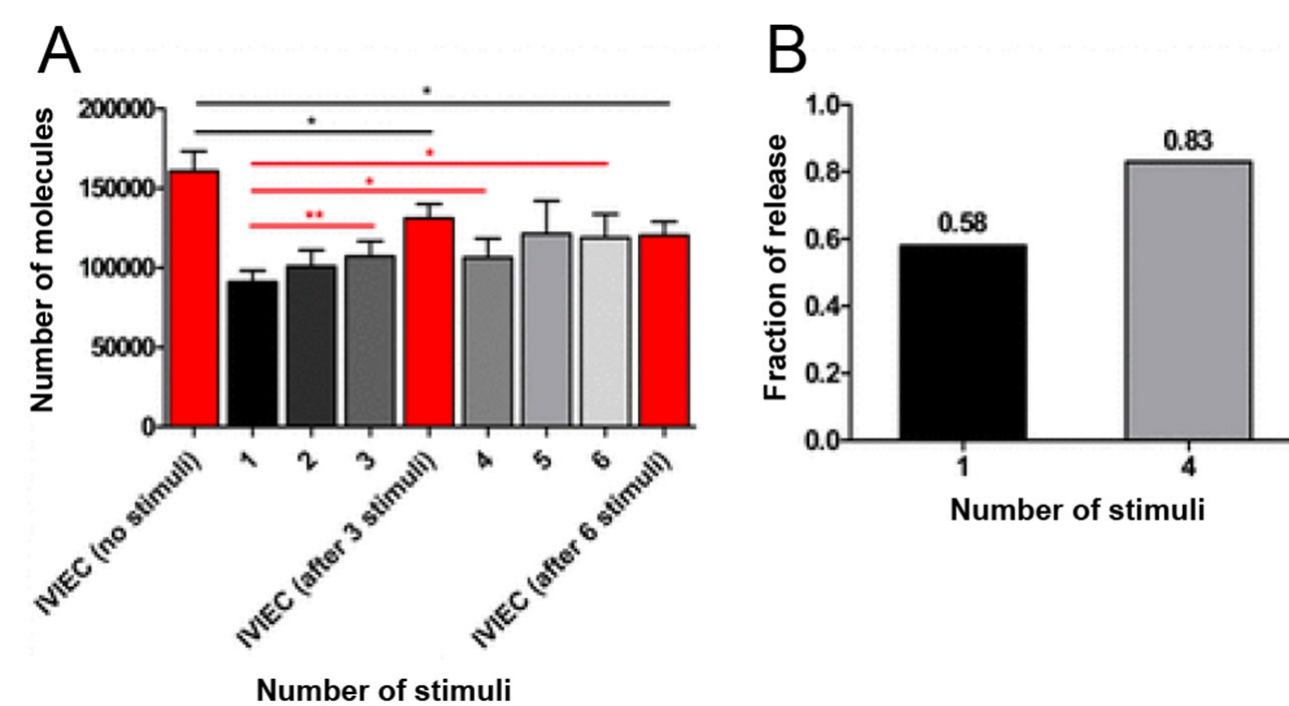
The amount of exocytotic release, vesicular content, and fraction of release during repetitive stimulations. A) Comparison of SCA for the sixth stimulation with IVIEC without stimulation and after the third and sixth stimulation. B) Fraction of release calculated for the first and fourth stimulation. Adapted with permission from ref. [42]. Copyright: 2019, National Academy of Sciences.

Short‐term synaptic plasticity in exocytosis was examined by repetitive stimulation in PC12 cells. The number of catecholamine events during exocytosis was decreased with six consecutive repetitive stimulations at 2 minute intervals (Figure 3).^[40]^ A decrease of calcium level after the six stimulations was also overserved. Exocytotic release was enhanced for many of the stimulus sequences. The vesicular catecholamine content decreased with short‐interval repetitive stimulations. Here, 161 000 molecules were detected before stimulation by IVIEC, while only 120 000 molecules were detected after the sixth stimulation. In these experiments, the fraction of release was 58 % for the first stimulation and increased to 83 % for the fourth stimulation, a molecular‐cellular memory effect. The paradigm here can be used to study cellular and exocytotic changes that might lead to the molecular initiation of short‐term memory.

There has also been an interest to investigate the side effects that are caused by anticancer drugs on exocytosis. Cisplatin, a cancer drug with memory impairment as a common side effect, has a dose‐dependent effect on the frequency and pore opening during exocytosis,^[41]^ leading to the investigation of other cancer drugs. Tamoxifen is used to treat estrogen receptor‐positive breast cancers, but causes memory and cognitive dysfunction. Tamoxifen treatment has a stimulatory effect at nanomolar level by increasing both exocytotic catecholamine release and vesicular content.^[42]^ However, at the micromolar level, tamoxifen has the opposite effect by decreasing the number of molecules during exocytosis and those stored in vesicles. The concentration of tamoxifen in the human brain during treatment is about 2–10 μM or higher at steady state. Thus, the different effects of tamoxifen on exocytosis and catecholamine storage in vesicles might be helpful to explain the side effect of memory dysfunction.

Dimethyl sulfoxide is a polar aprotic solvent that is extensively used as a vehicle for drug therapy in biological studies. DMSO has a strong solubility characteristic, however, it should be compatible with the growth medium with minimal toxic effect on cells. In a controversial set of experiments, DMSO treatment was shown to alter exocytosis by increasing the number of molecules released while having no effect on vesicular content.^[43]^ A level of 0.4 % of DMSO significantly increased exocytotic release of catecholamine and a greater increase was observed at 0.6 %. Higher concentrations of DMSO, such as 0.8 and 1 %, showed diminished effects on exocytosis but the amount of release was significantly higher than that of the control cells. Therefore, suitable concentration of DMSO and control experiments should be considered when considered as a vehicle to increase the solubility of drugs in cell biology experiments.

### 3.2 Octopamine exocytosis and vesicle content in *Drosophila melanogaster*

*Drosophila melanogaster*, the fruit fly, is a popular model organism to study biological processes related to human cognitive and neurodegenerative diseases. This is due to its short life span and amenable to genetic manipulation. Our group has studied *Drosophila* larvae and octopamine neurotransmitter release from exocytosis events at individual varicosities.^[44]^ Octopamine release from exocytosis was stimulated using optogenetics by a blue light, where octopaminergic terminals were labeled with the red fluorescent marker, mCherry, and the light sensitive ion channel channelrhodopsin‐2. About 23 000 octopamine molecules were shown to be released during exocytosis. Different amperometric spikes were observed, where flickering of release suggests partial release is the main mechanism of exocytotic octopamine release.

**Figure 4:**
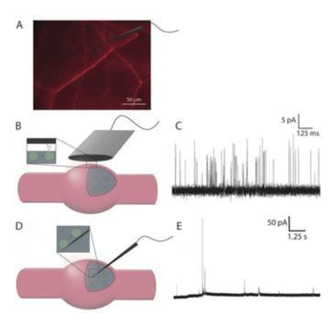
Detection of exocytotic release and vesicular content at a neuronal varicosity of *Drosophila* larva. A) Fluorescence microscopy image of mCherry‐labeled neurons in *Drosophila* larva showing the varicosities and a schematic of a nanotip electrode placed on or in a varicosity. B) Schematic of an electrode on a varicosity. C) Representative exocytosis current traces. D) Schematic of a nanotip electrode placed in the varicosity for IVIEC. E) Representative current traces for intracellular vesicle content measurements. Reprinted with permission from ref. [45]. Copyright: 2020, Wiley‐VCH.

In a study designed to look for partial release in fly neurons, intracellular quantification of octopamine in living *Drosophila* larval varicosities has been carried out with IVIEC (Figure 4).^[45]^ The average total content measured with IVIEC was 441 000 molecules per vesicle. Compared to the numbers of molecules released during simple or complex exocytosis events (20 000 and 47 000 molecules, respectively), the percentage of molecules released during exocytosis was only 4.5 % for simple events and 10.7 % for complex events. This further supports the concept of partial release in exocytosis and suggests it is considerably smaller in neurons than in neuroendocrine‐derived cells. The authors speculate that nerve cells only release a very small fraction of neurotransmitters during exocytosis allowing a greater range of regulation.

## 4 Conclusions and Future Directions

Electrochemical monitoring of exocytosis by combining single‐cell amperometry and intracellular electrochemical cytometry provides an analytical framework to examine the hypothesis often referenced to Katz that release is an all‐or‐none process. The evidence shows that release of catecholamines is predominantly partial with an open and closed vesicular event occurring. The effect of different chemical treatments on exocytosis and vesicular storage are summarized in Table 1. An extremely interesting set of results shows that drugs and ions like zinc can affect the fraction of released messenger during exocytosis, providing information potentially useful in understanding drugs of abuse and learning and memory.[^31^, ^32^] Overall, drugs that are thought to aid cognitive ability result in a higher fraction released and those that inhibit it result in a lower fraction released. In an experiment designed to develop plasticity, repeated stimulations of cells led to an increase in fraction released as measured by amperometry and IVIEC.^[40]^ These experiments are significantly advancing our knowledge of vesicular release and storage. The evidence from several studies now points partial release as the primary mechanism for release in many if not most cells. The partial release mechanism is thought to be the important part of the regulation of individual vesicular events and synaptic strength, especially in plasticity and cognitive, memory.

**Table 1:** Effects of different chemical and pharmacological treatments on exocytosis and vesicular content. Abbreviation used: NC, no change; N/A, not available.

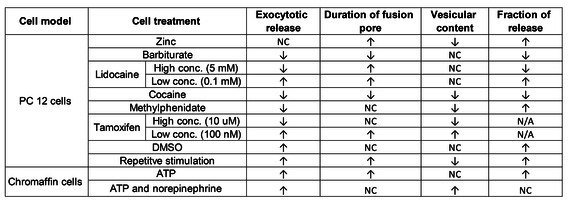

Data obtained from different cells and *Drosophila* larval varicosities for octopamine release convincingly suggest that partial release is the primary release mechanism of exocytosis. Similar results have been shown for release at synapses by the Huang and Amatore groups.[^46^, ^47^] If these experiments are representative, it is very likely that many transmitters are released by partial release. Preliminary evidence with a glutamate biosensor suggests partial release might occur for this transmitter as well.^[48]^ The number of glutamate molecules in an isolated vesicle of rodent brain was about 8 000, while only 5 200 molecules were released during exocytosis, resulting in 65 % fraction of glutamate release; however, control experiments blocking glutamate and without glutamate dehydrogenase are needed to give confidence that glutamate is indeed being measured in these experiments, leaving this still as an area to explore and verify.

Peptide hormones are also stored in large dense‐core vesicles that release their content during exocytosis. Previous imaging studies have shown that the fusion pore of insulin vesicle closes before the dense core content can be fully dispersed in some cases, but most insulin vesicles release all of their content during a single round of exocytosis.^[49]^ Future studies, using insulin biosensors are needed to further understand the release mechanism of insulin in various types of peptidergic endocrine cells. In addition, it would be also interesting to determine if a similar release mechanism is present for different neurotransmitters and hormones in different cells, especially single mammalian neurons, to shed light on unanswered questions in exocytosis.

## Conflict of interest

The authors declare no conflict of interest.

## Biographical Information

*Andrew Ewing is Professor of Chemistry and Molecular Biology at the University of Gothenburg (Sweden) and is currently also Academician Professor at Shenzhen University (P.R. China). He is a Knut and Alice Wallenberg Scholar (2011–2023), an elected member of the Royal Swedish Academy of Sciences, class 4 (chemistry), Nobel Class (2012), and the Gothenburg Academy of Arts and Sciences (2013). His research focuses on the neuronal process of exocytosis, pioneering small‐volume chemical measurements at single cells and the contents of individual nanometer vesicles in cells*.

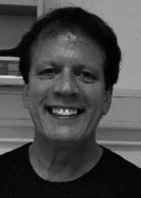

## Biographical Information

*Ying Wang is a postdoctoral researcher in Andrew Ewing's lab at the University of Gothenburg (Sweden). She received her Ph.D in analytical chemistry in 2018 from the University of Virginia, working under the supervision of Dr. Jill Venton. Her research interests focus on measuring dynamic chemical events during exocytosis and vesicular content at single‐cell levels using nano‐analytical approaches, as well as electrochemical detection of neurotransmitter release in animal models and the development of novel microelectrodes*.